# Antiresorptive effect of a semisynthetic triterpene from *Combretum leprosum* Mart. on dexamethasone-induced osteoporosis in rats

**DOI:** 10.1590/1414-431X2025e14833

**Published:** 2025-10-17

**Authors:** L.R. Garcez, P. Goes, J.L.T. Cavalcante, S.A. da Costa, W.L.C. Ribeiro, K.M.A. Pereira, F.W.G. Costa, P.G.B. Silva, F.S.R. Carvalho, C.J.A. Silva-Filho, A.L.O. Capistrano, M. Rauner, S. Thiele, F.G. Barbosa, J. Mafezoli, H.V. Chaves, R.F.C. Leitão, R.F. de Vasconcelos, M.M. Bezerra

**Affiliations:** 1 Programa de Pós-Graduação em Ciências da Saúde, Faculdade de Medicina, Universidade Federal do Ceará, Sobral, CE, Brasil; 2 Departamento de Patologia e Medicina Legal, Faculdade de Medicina, Universidade Federal do Ceará, Fortaleza, CE, Brasil; 3 Departamento de Morfologia, Faculdade de Medicina, Universidade Federal do Ceará, Fortaleza, CE, Brasil; 4 Departamento de Fisiologia e Farmacologia, Faculdade de Medicina, Universidade Federal do Ceará, Fortaleza, CE, Brasil; 5 Programa de Pós-Graduação em Ciências Morfológicas, Departamento de Morfologia, Faculdade de Medicina, Universidade Federal do Ceará, Fortaleza, CE, Brasil; 6 Programa de Pós-Graduação em Odontologia, Faculdade de Odontologia, Universidade Federal do Ceará, Fortaleza, CE, Brasil; 7 Departamento de Química Orgânica e Inorgânica, Centro de Ciências, Universidade Federal do Ceará, Fortaleza, CE, Brasil; 8 Division of Endocrinology, Diabetes and Bone Diseases, Department of Medicine III & Center for Healthy Aging, Dresden University of Technology, Dresden, Germany; 9 Instituto Federal do Ceará, Fortaleza, CE, Brasil

**Keywords:** *Combretum leprosum* Mart., Semisynthetic, Glucocorticoid, Osteoporosis, Rats

## Abstract

Chronic use of glucocorticoids is one of the most common causes of osteoporosis. Triterpenes have a positive effect on bone metabolism, which encourages research into the anti-resorptive properties of these natural compounds. In this study, the anti-resorptive effect of the semisynthetic compound 3β,6β,16β-tripropionyloxylup-20(29)-ene (CL-P2), obtained from the natural lupane-type triterpene 3β,6β,16β-trihydroxylup-20(29)-ene (CL-1) isolated from *Combretum leprosum* Mart., was investigated in a glucocorticoid-induced osteoporosis (GIO) model in rats. GIO was induced by dexamethasone (7 mg/kg, *im*; 1×/week, 65 days). On the 36th day, treatment was started (gavage) with CL-P2 (0.01 or 0.1 mg/kg; 30 days). After this time interval, the rats were euthanized and the femurs and lumbar vertebrae were collected for analysis by microcomputed tomography (micro-CT), quantity and type of collagen (Picrosirius Red), micro-Raman spectrometry, and histomorphometric analysis using hematoxylin and eosin (H&E) staining. The organs were collected for toxicity analysis (HE). CL-P2 increased trabecular volume, number of trabeculae and bone mineral density as evidenced by micro-CT analysis in the third lumbar vertebra (L3), as well as the amount of total collagen and type I collagen in L2. In the analysis of the femurs, CL-P2 promoted an increase in the number of osteoblasts and osteocytes and a reduction in the number of osteoclasts, as well as change in the mineral composition of these bones, suggested by the increase in the carbonate-to-phosphate ratio (CTPR) identified by micro-Raman spectrometry. Histopathological analysis of the organs revealed the pre-clinical safety of CL-P2. CL-P2 had bone-protective benefits and may be a biotechnologically viable product as a supplemental therapy for GIO.

## Introduction

Osteoporosis is an osteometabolic disease characterized by a combination of low peak bone mass, accelerated bone resorption, and decreased bone production with an increased risk of fractures, especially in the vertebrae, distal radius, and proximal femur, increasing morbidity and mortality rates (1).

Osteoporosis is classified as primary and secondary, with glucocorticoid-induced osteoporosis (GIO) being the most common type of secondary osteoporosis. Although glucocorticoids are one of the most prescribed drugs to treat various inflammatory and autoimmune diseases, prolonged exposure to therapeutic doses has damaging effects on bone cells (osteoblasts, osteoclasts, and osteocytes), affecting the balance between bone formation and resorption, with a decrease in bone density and changes in bone microarchitecture (2).

Osteoporosis treatment includes non-pharmacological (regular exercise, fall prevention program, intake of dairy products) and pharmacological measures (3). The latter include the combination of antiresorptive drugs (bisphosphonates, selective estrogen receptor modulators (SERMs), denosumab, calcium, and vitamin D supplementation) with anabolic drugs (teriparatide, strontium ranelate, and romosozumab) (4). However, despite the proven efficacy of these drugs, their long-term use is associated with the occurrence of side effects such as thrombosis, atherosclerosis, and hypertension (5), motivating pre-clinical and clinical research on options with a better risk/benefit ratio. This scenario encourages the search for new therapeutic options and compounds.

**Figure 1:**
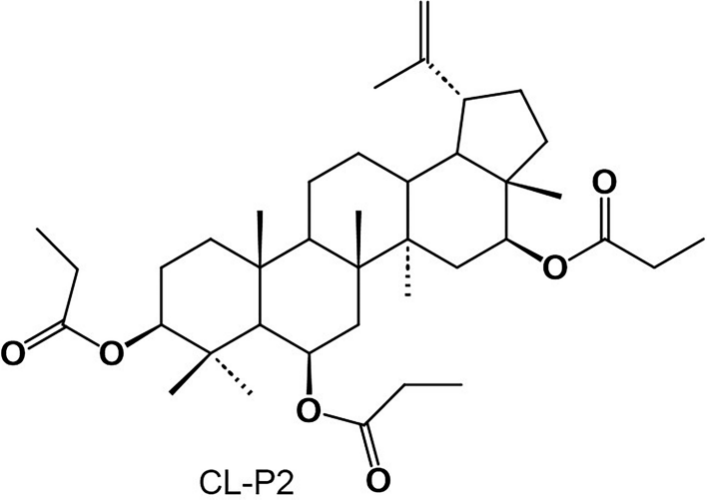
Chemical structure of the semisynthetic triterpene CL-P2.

*Combretum leprosum* Mart. (Combretaceae), popularly known as “mofumbo”, is a species distributed in the semi-arid *caatinga* of northeastern Brazil, which is widely used in folk medicine for its antimicrobial, antitumor, anti-ophidic, anti-hemorrhagic, and anti-inflammatory activities (6). Facundo et al. (7), in phytochemical studies in species of the genus *Combretum*, demonstrated the presence of several classes of bioactive compounds, including the triterpene 3β,6β,16β-trihydroxylup-20(29)-ene (CL-1), which has demonstrated a variety of biological actions, including antimicrobial (8), antinociceptive (9), and anti-inflammatory activity (10). Our research group produced from CL-1 a semisynthetic derivative 3β,6β,16β-tripropionyloxylup-20(29)-ene, named CL-P2 (Figure 1), which showed antinociceptive and anti-inflammatory effects and no toxicity when administered to mice for 14 days (11).

Considering that GIO is the most common cause of secondary osteoporosis and yet remains an undertreated condition, the reported biological activity of *Combretum leprosum* Mart., and the positive effect of triterpenes on bone metabolism (12), this study aimed to investigate the possible antiresorptive effect of the semisynthetic triterpene CL-P2 in the GIO model in *Wistar* rats.

## Material and Methods

### Obtaining the CL-P2 derivative

The semisynthetic derivative CL-P2 was obtained from the acylation reaction of the triterpene CL-1, isolated from the inflorescences of *Combretum leprosum* Mart. For the acylation reaction, 0.218 mmol (100 mg) of CL-1 was dissolved in 2.2 mL of dry CH_2_Cl_2_, together with 0.22 mmol (26.59 mg) of DMAP (4-dimethylaminopyridine), in a 25-mL flask. Next, 1.041 mmol (91 µL) of C_3_H_5_OCl (propanoyl chloride) and 0.22 mmol (31 µL) of Et_3_N (triethylamine) were added. The reaction was kept under constant stirring at room temperature and monitored by TLC (thin-layer chromatography) for 10 hours. After the reaction was completed, the mixture was concentrated under reduced pressure in a rotary evaporator and the mass obtained (231 mg) was purified by flash chromatography (column diameter=1.5 cm; silica=13 g) using hexane/ethyl acetate/methanol (9;0.5;0.5) as the eluent. Thus, the triacylated product CL-P2 was obtained in the form of a colorless resinous material with a 57.80% yield.

### Semisynthetic derivative CL-P2

The CL-P2 compound is a colorless resin with the following spectrometric data: ^13^C NMR (CDCl_3_, 75 MHz): δ 12.82 (C-28), 16.30 (C-27), 16.67 (C-26), 17.60 (C-24), 17.70 (C-25), 19.37 (C30), 21.11 (C-11), 23.92 (C-12), 24.82 (C-2), 27.60 (C-23), 29.81 (C-21), 33.66 (C-10), 36.76 (C-13), 36.96 (C-15), 37.69 (C-22), 37.98 (C-4), 38.65 (C-8), 40.06 (C-1), 40.42 (C-7), 44.41 (C-14), 47.48 (C-17), 47.63 (C-18), 47.94 (C19), 50.34 (C-9), 55.03 (C-5), 70.42 (C-6), 78.52 (C-16), 80.34 (C-3), 110.13 (C-29), 149.82 (C-20), 9.09, 28,11, 173.59 (propanoyl), 9.40, 28.14, 174.03 (propanoyl), 9.42, 28.65, 174.21 (propanoyl); ^1^H NMR (CDCl_3_, 300 MHz): δ 0.81 (s, CH_3_), 0.87 (s, CH_3_), 0.99 (s, CH_3_), 1.00 (s, CH_3_), 1.13 (9H, m, propanoyl), 1.20 (s, CH_3_), 1.21 (s, CH_3_), 1.66 (3H, s, H-30), 2.30 (6H, m, propanoyl), 4.41 (1H, dd, *J*=11.3 and 4.5 Hz, H-16), 4.59 (1H, sl, H-29), 4.69 (1H, sl, H-29), 4.83 (1H, dd, *J*=9.5 and 4.5 Hz, H-3), 5.51 (1H, sl, H-6); υ_max_ 3070, 2943, 1735, 1642 cm^-1^; (+)-HR-ESI-MS *m/z* 649.44312 [M+Na]^+^ (calculated for C_39_H_62_O_6_Na, 649.89579).

### Animals and study design

After approval by the Ethics Committee for Animal Use of the Federal University of Ceará (UFC), under protocol number 6655221121, 24 male Wistar rats aged between 10 to 12 weeks, weighing an average of 200 g, were obtained from Central Animal Facility of the Federal University of Ceará (UFC-Fortaleza, Brazil). Animals were kept in pairs in a cage, in a temperature-controlled environment (23±2°C), with a 12-h light/dark cycle and free access to water and food. The sample size of 8 animals per group was determined assuming an α=0.05 and a power of 0.8. The study was conducted in accordance with the ARRIVE guidelines (Animal Research: Report of *in vivo* experiments) and the guidelines of the Brazilian Society of Animal Science.

**Figure 2:**
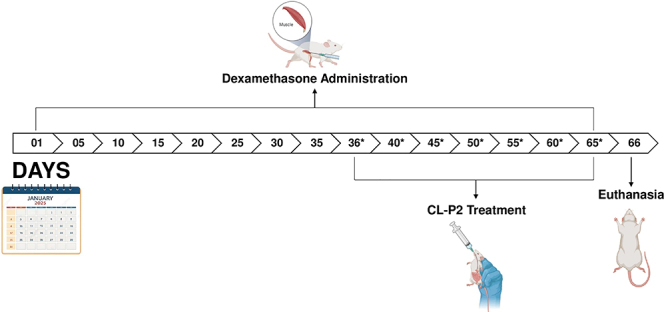
Schematic representation of the experimental timeline. Glucocorticoid-induced osteoporosis (GIO) was established by weekly intramuscular administration of dexamethasone (7 mg/kg) from day 1 to day 65. CL-P2 treatment (0.01 or 0.1 mg/kg, orally) was initiated on day 36 and continued daily for 30 days (indicated by asterisks). On day 66, all animals were euthanized, and biological samples were collected for analysis.

After two weeks of acclimatization, the animals were randomly divided into three groups: 1) GIO; 2) CL-P2 0.01 mg/kg; and 3) CL-P2 0.1 mg/kg. GIO was induced in all animals by adapting previously published protocols (3,13). Briefly, 7 mg/kg dexamethasone (Decadron^®^, 4 mg/mL; Ache, Brazil) was injected (*im*) every 7 days for 65 days. From day 36 onwards, groups 2 and 3 were treated (gavage) with 0.01 or 0.1 mg/kg CL-P2, respectively, for 30 consecutive days. Group 1 received 0.9% saline solution by gavage for the same period. The experimental timeline is illustrated in Figure 2.

All animals were euthanized on the 66th day by administering (*ip*) an overdose of ketamine and xylazine. The third lumbar vertebrae (L3) were collected for micro-CT analysis and the second lumbar vertebrae (L2) for analysis of the amount and type of collagen. The left femurs were also collected for histomorphometric analysis, and the right femurs for analysis by Raman spectroscopy. The organs (heart, stomach, liver, and kidneys) were collected for toxicity analysis.

### Computerized microtomography (micro-CT)

The L3 lumbar vertebrae were scanned using a high-resolution micro-CT system (vivaCT40, Scanco Medical, Switzerland). The scanning parameters were set to 70 kVp, 114 µA, 200 ms integration time, and voxel size of 10.5 µm^3^. One hundred slices around the center were analyzed. The trabecular compartment of the L3 vertebra was analyzed following Scanco Medical protocols. Bone volume (BV/TV), trabecular number (Tb.N), separation (Tb.Sp), and thickness (Tb.Th), as well as bone mineral density (BMD) were assessed (3). Data are reported as mean percentage±SE.

### Analysis of the amount and type of collagen

Previously prepared sections of L2 vertebral tissue were stained with Picrosirius red solution (ScyTek^®^, USA) and submitted to quantification analysis by conventional microscopy for total collagen and under polarized light for type I and type III collagen.

This study used photographs of 5 fields at 400× magnification. After calibrating the images, the areas of total collagen were identified using the Color Threshold command, adjusting the red, green, and blue color values in the RGB function. The images were then converted to an 8-bit color scale and binarized, and the percentage of the total collagen area was measured using the particle analysis command. The same protocol was applied to the polarized images, adjusting the color values to identify type I collagen (orange-red) and type III collagen (yellow-green). All the images were quantitatively evaluated using ImageJ^®^ software (NIH, USA) (14). The mean percentage of the slides was used as the sample unit, and the data are reported as mean percentage±SE.

### Micro-Raman spectroscopy

Bone composition and remodeling were assessed using micro-Raman spectrometry. Briefly, sectioned samples of the right femoral neck were placed in a micro-Raman spectrometer (XploRATM, Horiba Jobin Yvon, France) coupled to a confocal microscope (XploRATM model, Horiba JobinYvon). Three spectra were collected from each sample, acquired on the same day and in ambient conditions to avoid optical misalignment and changes in laser power, after calibrating the device with silica according to the manufacturer's instructions. Acquisitions were made at three different points on the femoral neck trabecular bone. The data were acquired using a data acquisition control system using the LabSpec 6 software (Horiba, Jobin Yvon) and analyzed using the Origin 9 program (Originlab^©^ Corporation, USA). To identify changes in bone tissue, the proportions of the following bands were calculated: 1) mineral-to-matrix ratio (MTMR) (∼960/1454 cm^-1^): quantifies bone mineralization; 2) carbonate-to-phosphate ratio (CTPR) (∼1070/∼960 cm^-1^): quantifies the extent of type B carbonate substitution, indicating tissue solubility; 3) HA carbonate/amide I (∼1.070/∼1.667 cm^-1^): evaluates bone remodeling (15). Data are reported as percentage mean±SE.

### Histomorphometric analysis

The left femurs were fixed in formaldehyde (10%) for 24 h and demineralized with formic acid (10%) for five days. To make the slides, the proximal femoral epiphysis was cut along the longitudinal axis, then dehydrated and embedded in paraffin, cut into 5-µm sections and prepared for hematoxylin and eosin (H&E) staining. Photos of the slides of five different fields of the femoral neck were taken at 400× magnification. An examiner who was unaware of the treatments carried out the histomorphometric assessment by counting the number of osteoblasts (N.Ob/B.Pm) and osteoclasts (N.Oc/B.Pm) per bone perimeter and the number of osteocytes per bone area (N.Oct/B.Ar). Cell counting was carried out using the ImageJ^®^ software using the cell contain command (15). Data are reported as percentage mean±SE.

### Toxicity analysis

The organs (heart, stomach, liver, and kidneys) were collected, fixed in buffered formalin (10%) for 24 h and processed for H&E staining.

An experienced pathologist (KMAP) blinded to the treatment groups performed the semi-quantitative analysis in the 5-µm sections, assigning scores ranging from 0 to 3 according to the severity of histopathological findings in the organs: kidneys (edema, congestion, hemorrhage, inflammatory infiltrate, necrosis), liver (congestion, hemorrhage, edema, inflammatory infiltrate), stomach (hemorrhage, edema, inflammatory infiltrate), and heart (hypertrophy, congestion/ hemorrhage, necrosis). The absence of these alterations in the organs under examination received a score of 0 (normal). For mild, moderate, and severe modifications, the corresponding scores were 1, 2, and 3, respectively, adapted from Ribeiro et al. (16).

### Statistical analysis

The Shapiro-Wilk test was used to test data normality. Parametric data are reported as means±SE and non-parametric data as medians (minimum and maximum). ANOVA followed by the Bonferroni test or the Kruskal-Wallis test followed by the Dunn test were used when appropriate. For all analyses, P<0.05 was considered statistically significant. Statistical analyses were carried out using GraphPad Prism 8 (USA) and SPSS version 20.0 for Windows^®^ (USA).

## Results

### Low-dose CL-P2 protected bone microarchitecture

**Figure 3:**
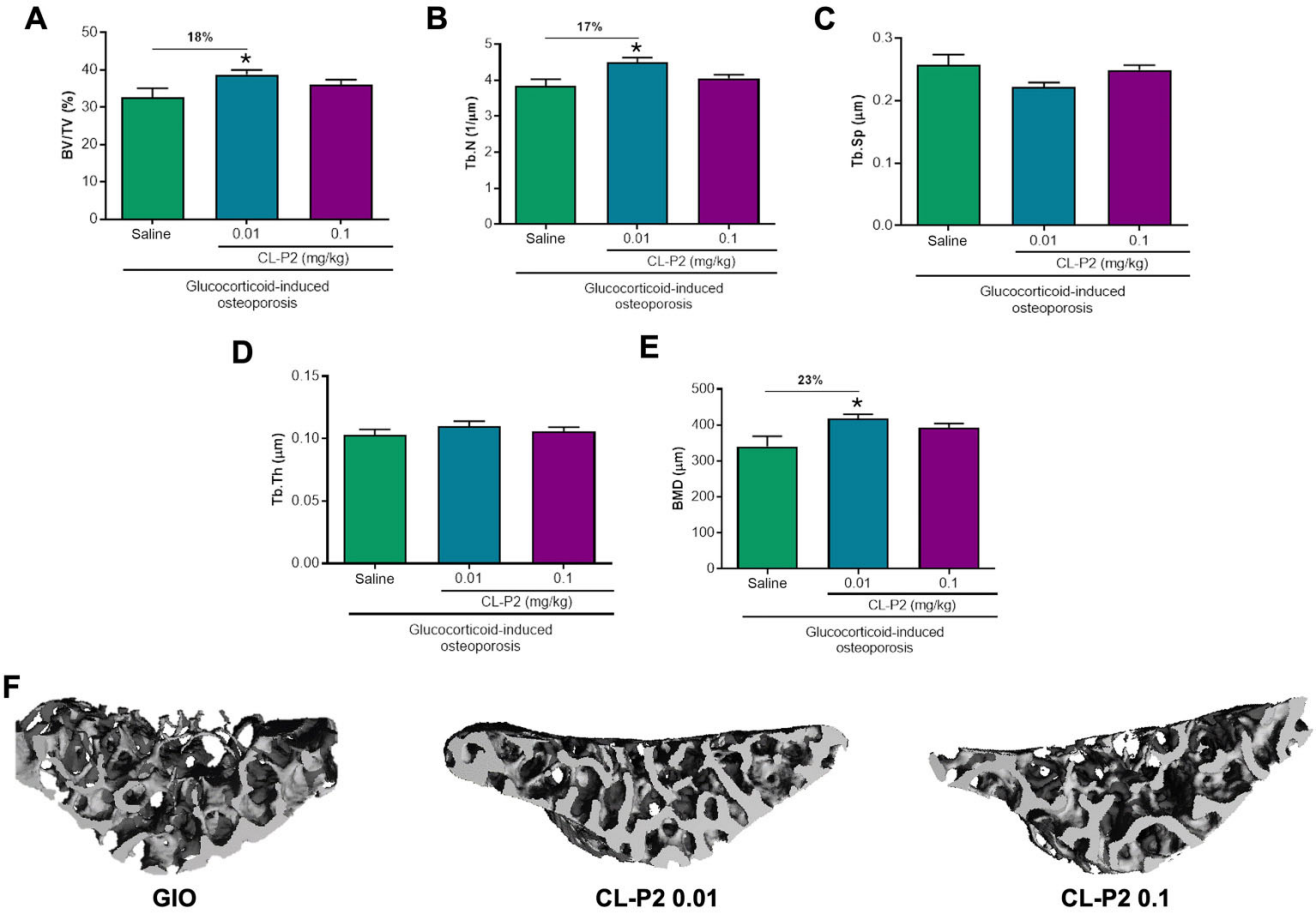
Micro-CT analysis of the third lumbar vertebra (L3) of rats with glucocorticoid-induced osteoporosis (GIO) and treated with saline or CL-P2 (0.01 or 0.1 mg/kg). **A**, Trabecular bone volume per total volume (BV/TV); **B**, trabecular number (Tb.N); **C**, trabecular separation (Tb.Sp); **D**, trabecular thickness (Tb.Th); (**E**) bone mineral density (BMD). Data are reported as means±SE of eight animals per group. *P<0.05, ANOVA followed by the Bonferroni test. **F**, Representative 3D images reconstructed from micro-CT analysis of the vertebral body of L3 of rats.

Micro-CT analysis revealed that CL-P2 reduced the lumbar vertebrae damage caused by dexamethasone. In fact, the group treated with CL-P2 (0.01 mg/kg) showed a significant increase (P<0.05) in trabecular volume (BV/TV) of 18% (Figure 3A) and trabecular number (Tb.N) of 17% (Figure 3B). In addition, the group treated with CL-P2 (0.01 mg/kg) showed a significantly (P<0.05) higher BMD of 23% (Figure 3E), compared to the untreated group. These findings are illustrated by the 3D images reconstructed from the micro-CT analysis of the L3 vertebral body (Figure 3F). No change was seen in the trabecular space (Tb.Sp) or thickness (Tb.Th) at the dose of 0.1 mg/kg CL-P2 (Figures 3C and D).

### CL-P2 improved bone quality

**Figure 4:**
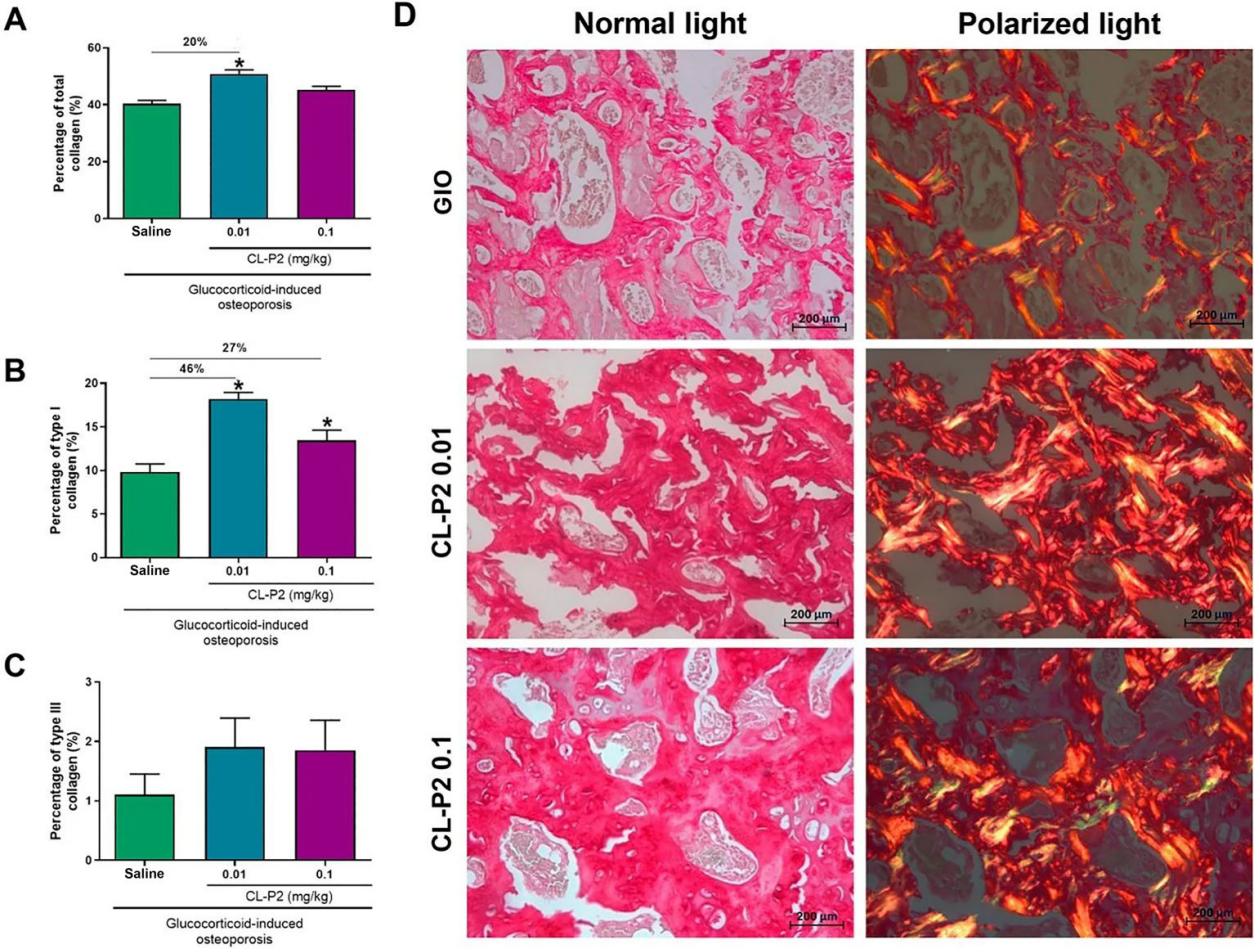
Effect of CL-P2 (0.01 or 0.1 mg/kg) or saline on the percentage of (**A**) total collagen, (**B**) type I collagen, and (**C**) type III collagen in rat vertebrae with glucocorticoid-induced osteoporosis (GIO). Data are reported as mean±SE of eight animals per group. *P<0.05, ANOVA followed by the Bonferroni test. **D**, Representative images of Picrosirius red staining of vertebrae under normal and polarized light (scale bar 200 μm, 400× magnification).

Bone quality was assessed using Picrosirius red staining and Raman spectroscopy. There was a significant increase (P<0.05) in the percentage of total collagen in the vertebrae of rats treated with CL-P2 (0.01 mg/kg) compared to the untreated group, with an increase of 20% (Figure 4A). Under polarized light, the groups treated with CL-P2 (0.01 and 0.1 mg/kg) showed an increase (P<0.05) in the amount of type I collagen of 46 and 27%, respectively compared to the untreated group (Figure 4B). As for type III collagen, there was no significant difference between the groups (Figure 4C). These findings are illustrated in the representative images of the histological aspects of Picrosirius red staining of the vertebrae of the untreated and CL-P2-treated groups (0.01 or 0.1 mg/kg) under normal and polarized light, respectively (Figure 4D).

Analysis of bone composition by Raman spectroscopy showed that treatment with CL-P2 (0.01 mg/kg) increased the CTPR compared to untreated animals, suggesting an increase in bone tissue quality. However, there was no change in MTMR and HA carbonate/amide I (bone remodeling) (Table 1).

**Table:** **Table 1.** Effect of CL-P2 on Raman analysis of femurs from rats with glucocorticoid-induced osteoporosis (GIO).

Parameters	Groups		
	GIO + saline	GIO + CL-P2 0.01	GIO + CL-P2 0.1
CTPR	0.27±0.02	0.39±0.05*	0.29±0.03
MTMR	7.42±1.8	4.97±1.39	3.15±0.65
Bone remodeling	2.37±0.31	1.81±0.22	1.48±0.15

GIO: animals receiving dexamethasone (*im*) and saline (gavage); GIO + CL-P2 0.01 and 0.1 mg/kg: animals receiving dexamethasone (*im*) and different doses of CL-P2 (gavage). CTPR: carbonate-to-phosphate ratio; MTMR: mineral-to-matrix ratio. Data are reported as means±SE. *P<0.05 compared to GIO + saline (ANOVA).

### CL-P2 stimulated bone formation

**Figure 5:**
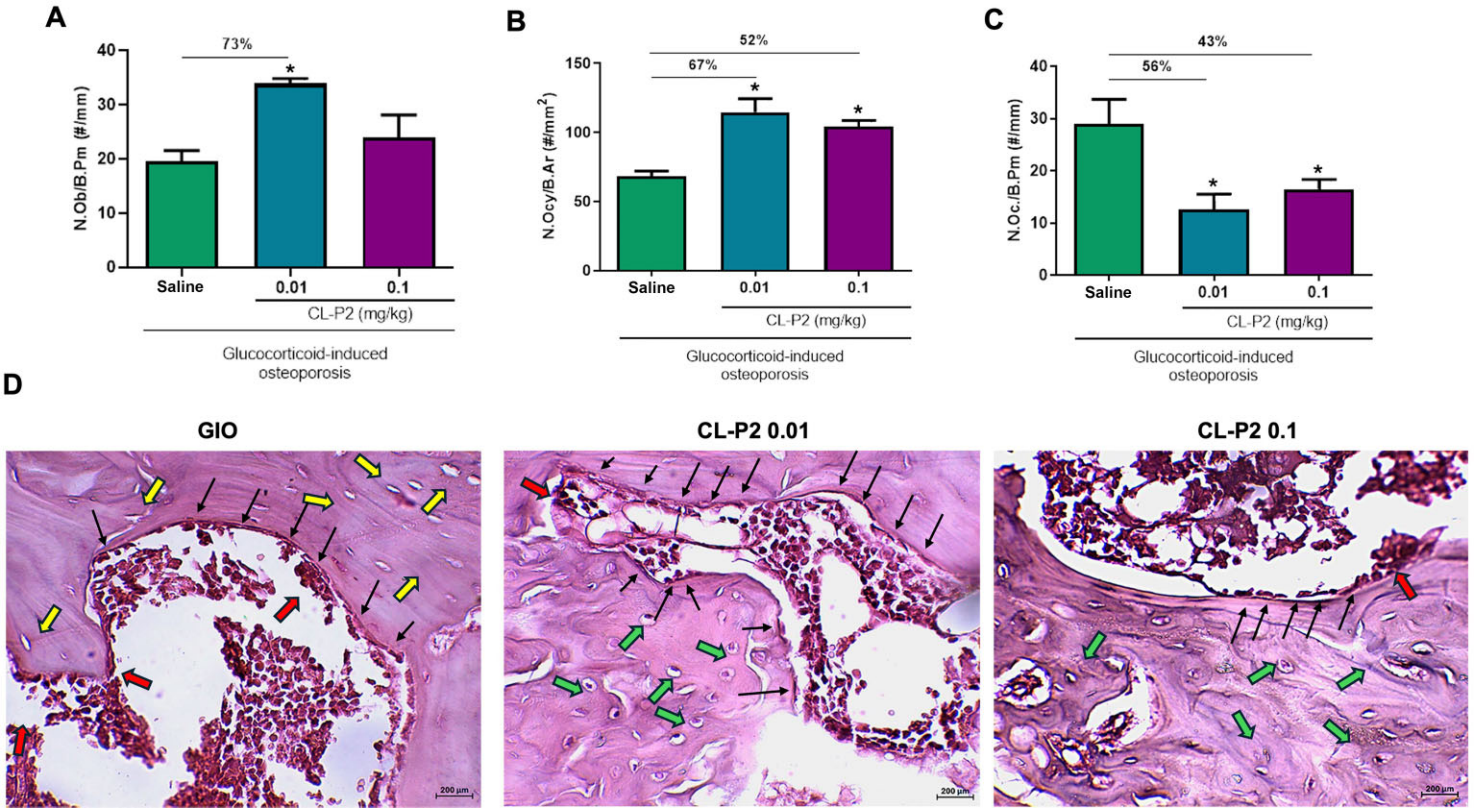
Effect of CL-P2 (0.01 and 0.1 mg/kg) or saline on the number of bone cells (osteoblasts, osteoclasts, and osteocytes) during glucocorticoid-induced osteoporosis (GIO) in rats. **A**, N.Ob./B.Pm (number of osteoblasts/bone perimeter); **B**, N.Oct/B.Ar (number of osteocytes/per bone area); and (**C**) N.Oc./B.Pm (number of osteoclasts/bone perimeter). Data are reported as means±SE. *P<0.05, ANOVA followed by the Bonferroni test. **D**, Representative images of hematoxylin and eosin (H&E) staining of femur bones. Black arrows indicate osteoblasts, green arrows, osteocytes, red arrows, osteoclasts, and yellow arrows, empty lacunae (scale bar 200 μm, 400× magnification).

When evaluating the effect of the treatment with CL-P2 (0.01 mg/kg) on the number of bone cells, a significant increase of 73% (P<0.05) was observed in the number of osteoblasts (Figure 5A) compared to the untreated group. Similarly, CL-P2 (0.01 and 0.1 mg/kg) showed a significant (P<0.05) increase of 67 and 52%, respectively, in the number of osteocytes compared to the untreated group (Figure 5B). In addition, the groups treated with CL-P2 (0.01 and 0.1 mg/kg) showed a significant reduction (P<0.05) of 56 and 43%, respectively, in the number of osteoclasts compared to the untreated group (Figure 5C). These quantitative findings are illustrated by representative H&E-stained histological images of femoral bone sections of the untreated (GIO) and CL-P2-treated groups (0.01 or 0.1 mg/kg) (Figure 5D).

### CL-P2 showed no toxicity

In the pre-clinical safety analysis, the administration (gavage) of CL-P2 (0.01 or 0.1 mg/kg) was not associated with the occurrence of deaths compared to the group that received only saline solution as treatment.

As for the histopathological (H&E) analysis of the organs, no significant microscopic findings were identified in the heart and stomach of the treated groups, compared to the untreated group (Table 2). Regarding the analysis of the liver and kidneys, reversible changes (edema and congestion) were observed between the groups (Table 2).

**Table 2:** Effect of CL-P2 treatment on histopathological analysis of the heart, stomach, liver, and kidneys of animals with glucocorticoid-induced osteoporosis (GIO).

Histopathological parameters	Groups		
	GIO+saline	GIO+CL-P2 0.01	GIO+CL-P2 0.1
Heart			
Hypertrophy	0 (0-1)	0 (0-1)	0 (0-1)
Congestion/bleeding	1 (0-2)	1 (0-2)	1 (0-1)
Necrosis	0 (0-0)	0 (0-0)	0 (0-0)
Stomach			
Bleeding	0.5 (0-1)	1 (0-1)	1 (0-1)
Edema	0 (0-1)	1 (0-1)	0.5 (0-1)
Inflammatory infiltrate	0 (0-0)	0 (0-1)	0 (0-1)
Liver			
Congestion	1 (0-2)	1 (0-2)	0 (0-1)*
Bleeding	1 (0-1)	0.5 (0-1)	0 (0-1)
Edema	2 (1-3)	1 (0-2)*	2 (1-2)
Inflammatory infiltrate	0 (0-0)	0 (0-0)	0 (0-0)
Kidneys			
Edema	0 (0-1)	1 (1-2)*	1 (1-2)*
Congestion	0 (0-1)	1 (0-1)	0.5 (0-2)
Bleeding	0 (0-1)	0 (0-0)	0 (0-1)
Inflammatory infiltrate	0 (0-0)	0 (0-0)	0 (0-0)
Necrosis	0 (0-0)	0 (0-1)	0 (0-1)

GIO + saline: animals that received dexamethasone (*im*) and saline (gavage); GIO + CL-P2 (0.01 and 0.1 mg/kg): animals that received dexamethasone (*im*) and CL-P2 (gavage) in their respective doses. Data are reported as median and range (n=8 for each treatment). *P<0.05 compared to the GIO + saline group (Kruskal-Wallis and Dunn's *post hoc* test).

## Discussion

The present study demonstrated both the antiresorptive activity and the preclinical safety of a semisynthetic derivative (CL-P2) obtained from *Combretum leprosum* Mart. in a GIO model in rats. CL-P2 enhanced BMD in the L3 vertebra, total and type I collagen in L2, and osteoblast and osteocyte numbers and decreased osteoclast numbers in the femurs. CL-P2 also changed the mineral composition, as shown by a higher CTPR assessed by Raman spectroscopy. In addition, the histopathological analysis of the organs revealed the pre-clinical safety of CL-P2. To our knowledge, this was the first demonstration of the use of a semisynthetic triterpene obtained from *C. leprosum* flowers in a GIO model in rats.

*C. leprosum* has great medicinal potential and different biological actions, including hepatoprotective, antinociceptive, anti-inflammatory, antibacterial, antioxidant, antibiofilm, and anticancer properties (6). Phytochemical studies have shown triterpenes to be one of the constituents of *C. leprosum* that may be responsible for its biological effects. Furthermore, triterpenes are among the main components found in medicinal plants that have been shown to improve bone damage in pre-clinical and clinical studies (17).

GIO is one of the most important side effects of using these drugs, leading to an increased risk of fracture (18). For a better understanding of the pathophysiology of GIO, as well as studying the efficacy and safety of new molecules for its treatment, animal models are extremely relevant. Rats and mice are the most frequently used species in GIO studies, and the use of DEX in rats is the most widely used model for studying GIO (19).

Under physiological conditions, there is a balance in the activities of osteoblasts and osteoclasts, keeping BMD within normal limits. However, during chronic bone diseases such as osteoporosis, this balance is disrupted (20). In the present study, a weekly injection (*im*) of DEX for 65 days led to changes in bone microarchitecture identified by micro-CT, which were reduced by treatment with CL-P2. Other authors using the micro-CT technique have demonstrated bone microarchitecture protection by triterpenes, including a significant increase in BMD in femoral trabecular bone (21).

Collagen is the most common structural protein found in humans and other mammals, and particularly the types I, II, and III account for more than 90% of total body collagen (22). In the present study, treatment with CL-P2 significantly increased the amount of total collagen compared to the untreated group. The triterpene 3β,6β,16β-trihydroxylup-20(29)-ene isolated from the leaves of *C. leprosum* stimulated collagen production in a wound healing model in mice (23). Also, our findings are in agreement with those of Hashim et al. (24) who showed that the extract obtained from the leaves of *Centella asiatica,* containing four different types of triterpenes, stimulated collagen synthesis in human dermal fibroblasts. This result was similar to that found by Morikawa et al. (25) who identified that the methanolic extract of *Bellis perennis* flowers (*Asteraceae*), containing oleanane-type triterpene saponins, promoted collagen synthesis in human dermal fibroblasts.

In the present study, the groups treated with CL-P2 had higher amounts of type I collagen than the untreated group. Some authors have shown that the DEX excess reduces the levels of type I collagen both *in vitro* and in mice submitted to GIO (26,27). The greater volume and number of trabeculae in L2, as well as the greater number of osteoblasts in the femurs observed after treatment with CL-P2 may contribute, at least partially, to the increase in collagen observed in the present study.

One of the main features of GIO is the direct effects on bone cells, including osteoblastogenesis suppression, reducing the number of osteoblasts and osteocytes, compromising bone formation (28). In the present study, the histomorphometric analysis of the femurs revealed a significant increase in the number of osteoblasts and osteocytes after CL-P2 administration. These findings are consistent with our earlier publication, in which our team of researchers showed that CL-P2 enhanced osteoblast activation and proliferation, hence revealing its osteogenic potential. These effects were caused by the modulation of the BMP-2, WNT, and RANK-L/OPG signaling pathways, which are important mediators of osteoclastogenesis (29). Our findings support those of Park et al. (30) who demonstrated the osteogenic effect of a triterpene obtained from the roots of *D. dasycarpus* through osteoblast differentiation. Given these findings, one can suggest that the increase in osteoblasts observed after treatment with CL-P2 may contribute to a better understanding of the therapeutic effects of this semisynthetic triterpene on bone, encouraging the development of new strategies for the treatment of osteoporosis and other diseases related to bone metabolism.

In the GIO model in rats, the enhancement of bone resorption is closely associated with an increased number of osteoclasts (31). In the present study, the groups treated with CL-P2 showed a significant reduction in the number of osteoclasts in the femurs compared to the untreated group. This finding has clinical significance, considering that osteoclasts are the main cells responsible for bone resorption, and during osteoporosis, their formation and function are increased (32). Furthermore, this finding becomes more relevant considering that, according to some authors, the anti-resorptive activity of triterpenes depends, at least partially, on reducing the number of osteoclasts. In this sense, the study by Koyama et al. (33) using the leaves of *Terminalia catappa L.*, a plant from the same family as *C. leprosum*, demonstrated suppressive effects on osteoclast differentiation in a model of osteoporosis caused by ovariectomy in mice. There are also reports in the literature of triterpenes from other families inhibiting osteoclast formation and bone resorption (34). In this sense, Zheng et al. (35) demonstrated that the pentacyclic triterpene ursolic acid blocked osteoclast differentiation and formation, thus attenuating ovariectomy-induced osteoporosis in rats. In addition, Wang et al. (36) demonstrated that cycloastragenol, a triterpene with antioxidant and anti-inflammatory activity, promoted bone repair by inhibiting osteoclasts in a model of glucocorticoid-induced osteonecrosis of the femoral head in rats. Thus, these findings suggest that triterpenes may be promising candidates for the treatment of osteoclast-related diseases, such as osteoporosis.

Raman spectroscopy is a fast and accurate technique for examining the composition of bone tissue. In the present study, Raman spectroscopy data of the CL-P2 group (0.01 mg/kg) revealed greater CTPR (carbonate HA>phosphate HA), thus characterizing the presence of an immature bone matrix in formation, corroborating the findings of the micro-CT analysis of this group (increased trabecular volume and number of trabeculae, increased BMD), compatible with a bone matrix in formation. Furthermore, these findings are consistent with the increase in cell activity observed in the present study (increased number of osteoblasts and osteocytes), corroborating the pattern of trabecular formation found in the micro-CT analysis and the formation of immature bone matrix in the Raman spectroscopy. In this sense, it has been shown that Raman spectroscopy can detect changes in the biochemical composition of bone aimed to analyze the effects of anti-osteoporotic treatments (37). In fact, data from the literature suggest that predictions of the degree of phosphate mineralization and overall mineral composition in the tibiae of mice in the rheumatoid arthritis model based on Raman spectrum measurements were as accurate as those produced by parameters derived from micro-CT (38).

As for the histopathological analysis, no changes were observed in the heart and stomach, while the liver and kidneys showed reversible changes. Although mild and reversible edema and congestion were observed in the kidneys of some treated animals, these findings were also present in the GIO + saline group, suggesting that such alterations are more likely associated with the chronic use of dexamethasone than with CL-P2 itself. Furthermore, the absence of necrosis or inflammatory infiltrate and the lack of a dose-dependent pattern indicate that CL-P2 did not induce renal toxicity under the conditions tested. Nevertheless, these findings highlight the need for further studies focusing on renal safety and clinical relevance, particularly with longer treatment periods. This finding is relevant considering the reports in the literature of liver and kidney toxicity associated with the use of herbal medicines for the treatment of osteoporosis (39).

Interestingly, the lower dose of CL-P2 (0.01 mg/kg) demonstrated superior outcomes compared to the higher dose (0.1 mg/kg) in several parameters, including bone mineral density, trabecular volume, and osteoblast/osteocyte numbers. Although a classical dose-dependent effect was not observed, this inverted U-shaped (hormetic) response has been described for other natural and semisynthetic compounds with osteogenic potential (40).

Although the GIO model in rats is validated, one of the limitations of this study was the use of only male rats, since osteoporosis is a serious health problem with impacts on both sexes. As a strength of the study, we can point out that bone loss was assessed via micro-CT, which allows a three-dimensional analysis and is precise in the study of osteoporosis. Furthermore, the study identified a therapeutic option for GIO, and this option was a triterpene, considering the already documented benefit of triterpenes in bone metabolism. Moreover, this triterpene is obtained from a semisynthesis, a powerful tool for pharmaceutical innovation.

## Conclusion

The results of this study showed that therapeutic administration of CL-P2 was safe and improved bone microarchitecture, and this effect can be credited, at least in part, to an increase in the number of osteoblasts and osteocytes and a reduction in osteoclasts. CL-P2 may exert protective effects on bone and represent a product with biotechnological potential as a complementary therapy for osteoporosis and fracture prevention in cases of prolonged glucocorticoid therapy. However, it is necessary to carry out further pre-clinical trials with further analysis, such as gene activity, to investigate the action mechanisms of CL-P2 in bone metabolism, and clinical trials to assess its use in humans.
